# White matter hyperintensity radiomics predicts 90-day diffusion-weighted imaging-confirmed acute ischemic stroke after diffusion-weighted imaging-negative transient ischemic attack: preliminary findings from a retrospective machine-learning study

**DOI:** 10.3389/fmed.2026.1913196

**Published:** 2026-09-16

**Authors:** Lian Liu, Yunyun Lyu, Zhiping Li, Yu Zhou

**Affiliations:** 1 Department of Medical Imaging, Jiangjin Hospital, Chongqing University of Chinese Medicine, Chongqing, China; 2 Department of Geriatrics, Jiangjin Hospital, Chongqing University of Chinese Medicine, Chongqing, China

**Keywords:** acute ischemic stroke, machine learning, radiomics, support vector machine, T2-fluid-attenuated inversion recovery, transient ischemic attack, white matter hyperintensities

## Abstract

**Objective:**

To develop and internally validate a T2-fluid-attenuated inversion recovery (T2-FLAIR) white matter hyperintensity (WMH) radiomics model, alone and in combination with baseline clinical variables, for predicting diffusion-weighted imaging (DWI)-confirmed acute ischemic stroke (AIS) within 90 days after an index DWI-negative transient ischemic attack (TIA).

**Methods:**

This single-center retrospective study included 398 patients initially diagnosed with DWI-negative TIA between January 2021 and May 2025 who underwent repeat brain magnetic resonance imaging with DWI within 90 days because of recurrent transient neurological symptoms and were classified into AIS (*n* = 125) and non-AIS (*n* = 273) groups according to follow-up DWI and apparent diffusion coefficient findings. Baseline WMH regions of interest were segmented on index T2-FLAIR images, and 1,781 radiomics features were extracted. Reproducible features were screened by least absolute shrinkage and selection operator regression, and 21 features were retained to calculate a radiomics score. Clinical, radiomics, and combined models were constructed using a support vector machine, with AIS defined as the positive class.

**Results:**

In the training cohort, the combined model achieved the highest area under the curve (AUC) (0.900, 95% CI: 0.860–0.940), followed by the radiomics model (0.862, 95% CI: 0.812–0.912) and the clinical model (0.775, 95% CI: 0.720–0.830). In the testing cohort, the radiomics model achieved the highest AUC (0.854, 95% CI: 0.757–0.950), followed by the combined model (0.841, 95% CI: 0.748–0.935) and the clinical model (0.696, 95% CI: 0.568–0.824).

**Conclusion:**

These preliminary findings indicate that T2-FLAIR WMH radiomics may provide promising internal discrimination for predicting 90-day DWI-confirmed AIS after DWI-negative TIA in this selected high-risk cohort. The incremental value of adding clinical variables to radiomics was not confirmed in the internal testing cohort, and prospective multicenter validation is required.

## 1 Introduction

Transient ischemic attack (TIA) is now generally defined using a tissue-based concept as a transient episode of focal neurological dysfunction caused by presumed cerebral, spinal, or retinal ischemia without acute infarction on neuroimaging (1) and represents both a neurological emergency and an important warning event for subsequent ischemic stroke. Despite transient symptoms, clinically relevant early stroke risk makes accurate risk stratification essential for timely diagnostic evaluation, secondary prevention, and resource allocation (2, 3).

Clinical prediction tools such as the ABCD2 score are widely used to estimate early stroke risk after TIA, but their discrimination is modest and they do not fully incorporate imaging biomarkers that may reflect underlying cerebrovascular vulnerability (4). Current secondary prevention guidelines emphasize that risk factor control, vascular evaluation, and etiology-directed treatment are central to reducing recurrent stroke after TIA or minor ischemic stroke (5). Therefore, imaging-derived markers that can identify patients at higher risk may complement conventional clinical scoring systems.

White matter hyperintensities (WMH) on T2-fluid-attenuated inversion recovery (T2-FLAIR) magnetic resonance imaging (MRI) are a common manifestation of cerebral small vessel disease and are associated with chronic ischemic injury, demyelination, gliosis, and impaired microvascular reserve (6). Prior studies suggest that WMH burden may be associated with TIA diagnostic certainty, post-TIA outcomes, and recurrent cerebrovascular risk (7, 8). White-matter microstructural alterations have also been associated with slower information processing in patients with metabolic syndrome, illustrating how clustered, clinically silent vascular risk factors may influence white-matter integrity and cognition during aging (9). However, conventional WMH assessment methods, including visual rating scales and volumetric measurements, may not capture intralesional heterogeneity, spatial distribution, and subtle signal-pattern information.

Radiomics converts medical images into high-dimensional quantitative features that characterize intensity distribution, shape, texture, and filtered image patterns (10). T2-FLAIR was selected because cerebrospinal-fluid suppression improves the conspicuity of periventricular and deep WMH, the sequence is routinely acquired without contrast in TIA MRI protocols, and it depicts chronic white-matter injury that complements a negative baseline DWI. In theory, T2-FLAIR WMH radiomics may provide information beyond visual burden by capturing microstructural and spatial heterogeneity within WMH. However, radiomics applications in TIA outcome prediction remain limited. Clinical translation is challenged by feature sensitivity to field strength, acquisition and reconstruction parameters, slice thickness, segmentation, preprocessing, and model-development choices, as well as by limited external validation, calibration, and interpretability; rigorous leakage prevention and transparent reporting are therefore essential (11–14).

This study aimed to develop and internally validate T2-FLAIR WMH radiomics-based machine-learning models for predicting DWI-confirmed AIS within 90 days after an index DWI-negative TIA and to evaluate whether combining clinical variables with radiomics improved predictive performance compared with clinical or radiomics features alone.

## 2 Materials and methods

### 2.1 Study design, population, and outcome definition

This retrospective single-center study screened 8,679 neurology inpatients who underwent brain MRI with DWI between January 2021 and May 2025. The study population was derived from patients with an index diagnosis of DWI-negative TIA who subsequently underwent repeat brain MRI with DWI within 90 days because of recurrent transient neurological symptoms. TIA was defined as transient focal neurological dysfunction caused by presumed cerebral or retinal ischemia without acute infarction on baseline DWI and apparent diffusion coefficient (ADC) imaging (1).

Inclusion criteria were as follows: (1) index admission with focal neurological symptoms or signs lasting less than 24 h; (2) absence of acute infarction on baseline DWI and ADC maps; and (3) repeat brain MRI with DWI within 90 days after the index event. Exclusion criteria were as follows: (1) coexisting lacunes of presumed vascular origin; (2) other cerebrovascular lesions, including cerebral aneurysm or vascular malformation; (3) malignant intracranial lesions; and (4) primary intracranial hemorrhage, including parenchymal hemorrhage or subarachnoid hemorrhage. After applying these criteria, 398 patients were included in the final analytic cohort (Figure 1).

**Figure 1:**
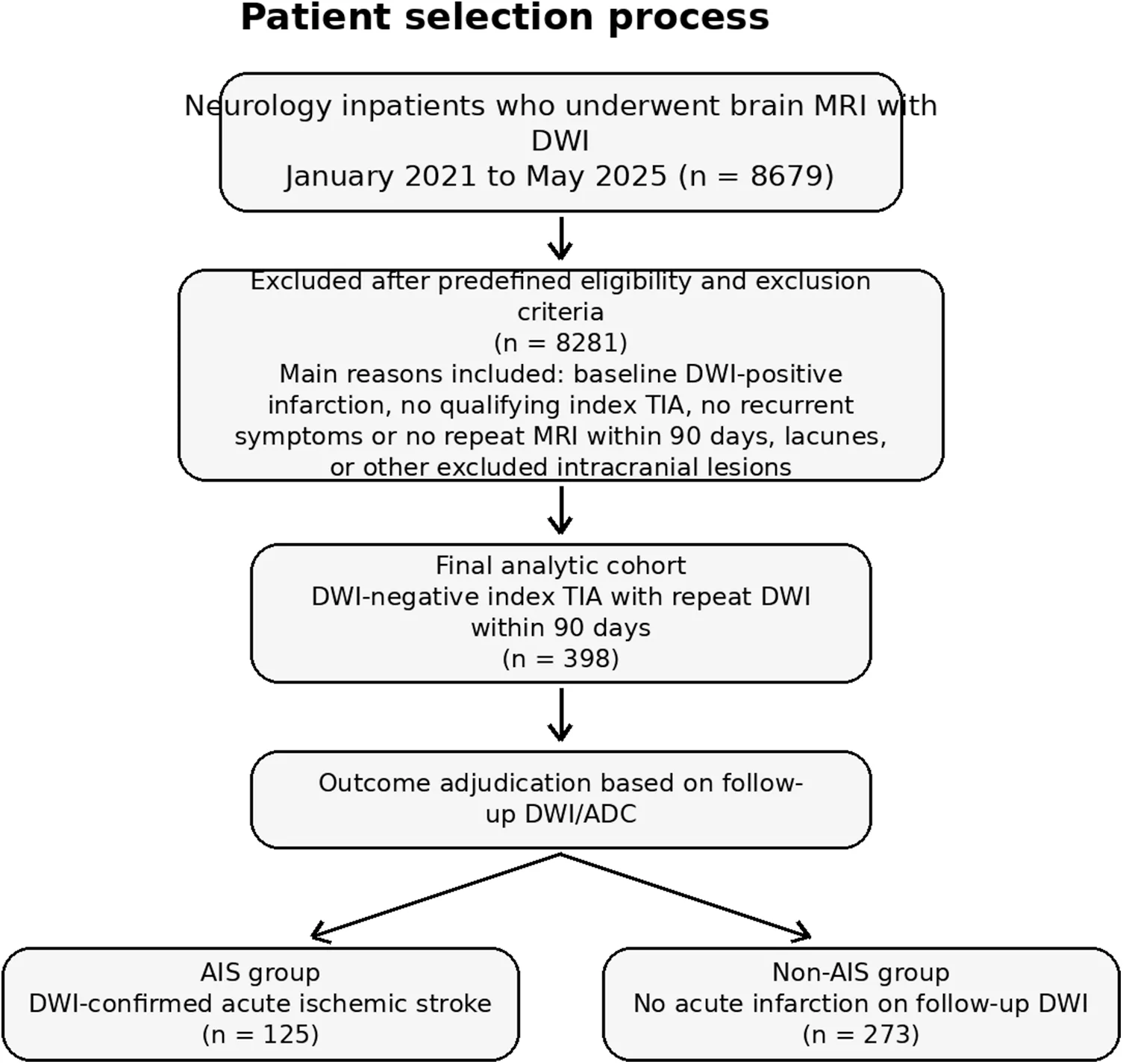
Patient selection process. The study screened 8,679 neurology inpatients who underwent brain magnetic resonance imaging with diffusion-weighted imaging between January 2021 and May 2025. After applying predefined eligibility and exclusion criteria, 398 diffusion-weighted imaging-negative transient ischemic attack patients with repeat diffusion-weighted imaging within 90 days were included; 125 were classified as acute ischemic stroke and 273 as non-acute ischemic stroke according to follow-up diffusion-weighted imaging/apparent diffusion coefficient findings.

The primary outcome was DWI-confirmed AIS within 90 days after the index TIA. Patients were assigned to the AIS group when repeat DWI showed a new acute ischemic lesion with corresponding ADC reduction. Patients with no acute infarction on follow-up DWI were assigned to the non-AIS group. Because the inclusion criteria required repeat MRI within 90 days, this cohort represents a selected higher-risk population with recurrent symptoms or clinical indication for repeat imaging; therefore, the findings should not be directly generalized to all patients with TIA.

A total of 125 patients were classified into the AIS group and 273 into the non-AIS group. Patients were randomly divided in an 8:2 ratio into a training cohort (*n* = 318) and a testing cohort (*n* = 80). According to the event distribution used for performance calculation, the training cohort included 100 AIS and 218 non-AIS patients, and the testing cohort included 25 AIS and 55 non-AIS patients.

Baseline clinical variables were extracted from the electronic health record system at the index TIA admission. These variables included age, sex, smoking history, alcohol consumption, coronary heart disease, hypertension, diabetes mellitus, systolic and diastolic blood pressure, fasting plasma glucose (FPG), total cholesterol (TC), triglycerides (TG), high-density lipoprotein (HDL), low-density lipoprotein (LDL), and ABCD2 score. Age was analyzed as a continuous candidate variable, and no study-specific age groups were created. The only age categorization was that embedded in the standard ABCD2 score (age ≥ 60 years = 1 point; age < 60 years = 0 points) (4). Post-TIA secondary prevention measures were not consistently available in the retrospective dataset and were therefore not included in the model; this issue is addressed as a limitation.

### 2.2 Image acquisition

All MRI examinations were performed using a GE Optima MR360 1.5 T scanner with patients positioned supine in a dedicated head coil. The standardized protocol included T1-weighted imaging [T1WI: repetition time [TR] 1,800 ms, echo time [TE] 20 ms], T2-weighted imaging (T2WI: TR 4,600 ms, TE 114 ms), T2-FLAIR (TR 8,500 ms, TE 153 ms), and DWI (TR 3,900 ms, TE 88 ms; b values = 0 and 1,000 s/mm²). All sequences were acquired in the axial plane from the vertex to the foramen magnum, with slice thickness of 6 mm, interslice gap of 1 mm, and matrix size of 256 × 256. Although 1.5 T provides lower signal-to-noise ratio and spatial resolution than 3 T for subtle tissue characterization, it is widely available and comparatively affordable in community and primary hospitals. Use of this routine platform therefore provides a pragmatic assessment of whether T2-FLAIR radiomics could be implemented in resource-limited settings, while cross-field-strength and cross-vendor validation remains necessary.

### 2.3 Region-of-interest segmentation and radiomics feature extraction

WMH regions of interest (ROIs) were segmented on baseline T2-FLAIR images obtained at the index TIA admission. Axial T2-FLAIR Digital Imaging and Communications in Medicine (DICOM) images were imported into the Darwin Research Platform (version 2.0). WMH ROIs were delineated slice by slice using semi-automated threshold-based segmentation followed by manual correction when necessary. Non-WMH hyperintensities, motion artifacts, chronic infarcts, enlarged perivascular spaces, and hemorrhagic or mass lesions were excluded from the ROI when identifiable.

To assess reproducibility, 30 randomly selected cases were independently segmented by two attending radiologists. Radiomics features extracted from the two sets of ROIs were compared using the intraclass correlation coefficient (ICC), and features with ICC > 0.75 were considered reproducible and retained for subsequent feature selection. Discrepancies in segmentation were resolved by consensus with a senior neuroradiologist.

A total of 1,781 radiomics features were extracted from each patient using PyRadiomics (version 3.1) through the Darwin Research Platform. Feature classes included first-order statistics, shape features, and texture features derived from gray-level co-occurrence matrix, gray-level run-length matrix, gray-level size-zone matrix, gray-level dependence matrix, and neighboring gray-tone difference matrix. Filter-derived features included local binary pattern, Laplacian of Gaussian, square, square root, gradient, exponential, logarithm, and wavelet transformations. Feature calculation followed Image Biomarker Standardisation Initiative principles where applicable (11). The platform-derived coefficient ranking of the final radiomics features is shown in Figure 2.

**Figure 2:**
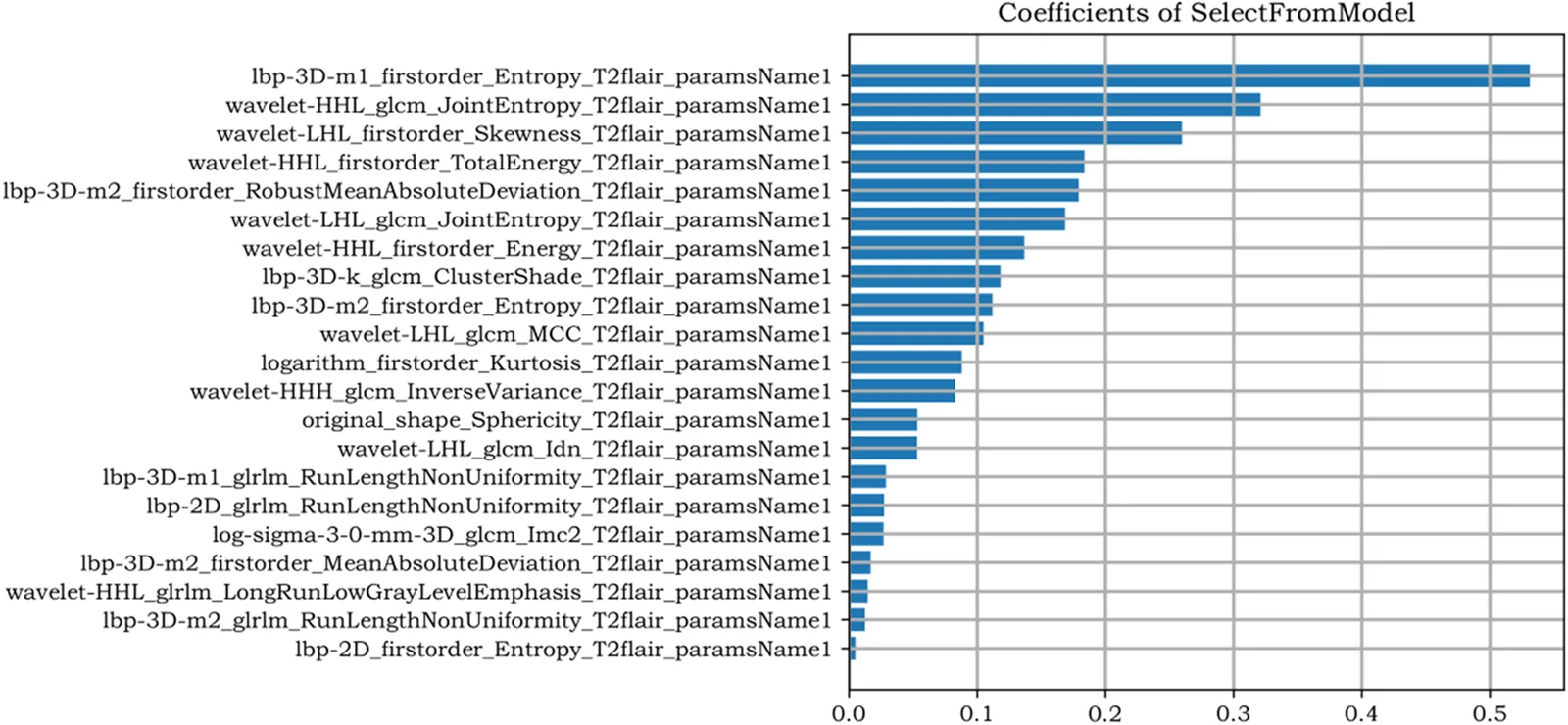
Platform-derived coefficient ranking of the 21 least absolute shrinkage and selection operator-selected radiomics features.

### 2.4 Feature selection and model development

To reduce data leakage, radiomics standardization, feature selection, and model fitting were restricted to the training cohort. Z-score standardization parameters were derived from the training cohort and then applied to the testing cohort. Least absolute shrinkage and selection operator (LASSO) regression with 10-fold cross-validation in the training cohort was used to select informative radiomics features. Twenty-one radiomics features were retained and linearly integrated into a radiomics score (rad-score).

Three support vector machine (SVM)-based binary classification models were constructed: a clinical model using selected baseline clinical variables, a radiomics model using the radiomics score or radiomics features, and a combined model incorporating both clinical and radiomics information. AIS was defined as the event of interest and positive class. Model output was evaluated in both the training and testing cohorts. The overall radiomics workflow is shown in Figure 3.

**Figure 3:**
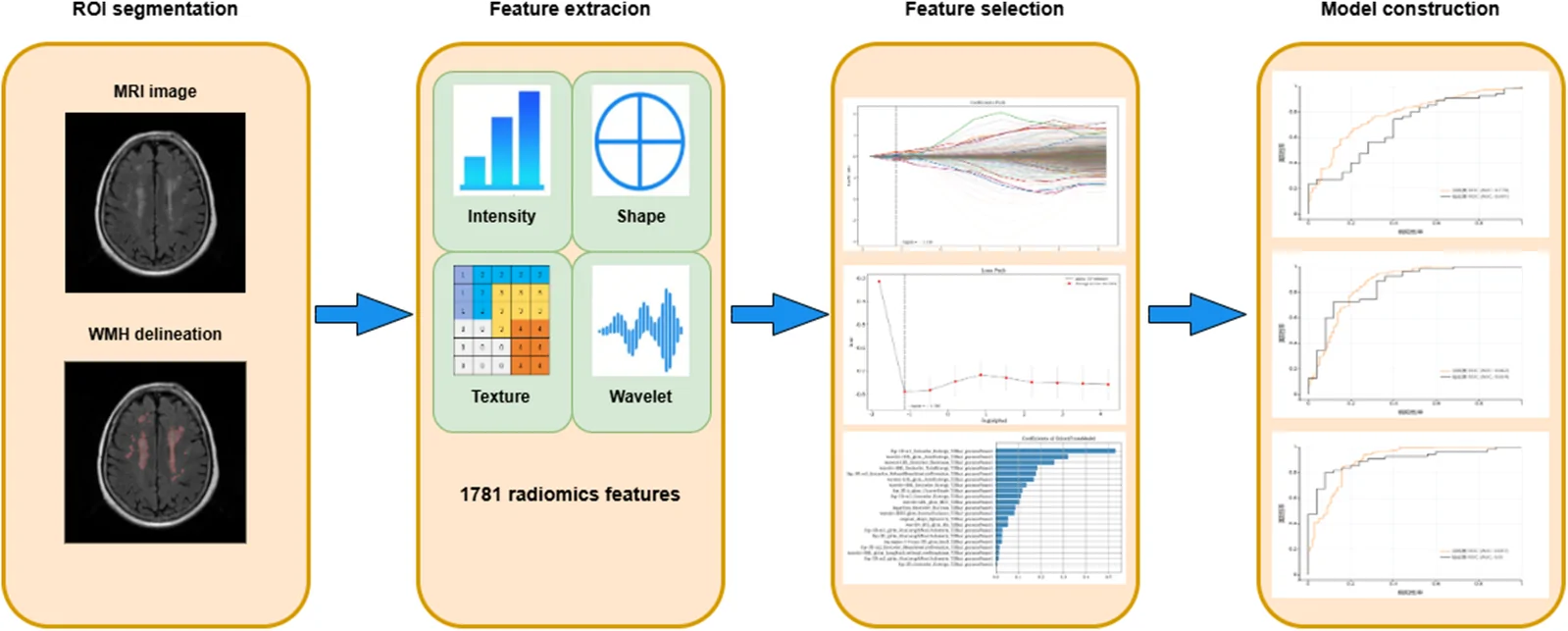
Radiomics workflow. Baseline T2-fluid-attenuated inversion recovery images were segmented to identify white matter hyperintensity regions of interest; radiomics features were extracted and selected; and clinical, radiomics, and combined machine-learning models were constructed and evaluated.

Because this was a retrospective platform-based analysis, full SVM hyperparameter settings, probability-calibration details, and random seeds were not available from the exported model report. These parameters should be prospectively recorded in future validation studies to enhance reproducibility.

### 2.5 Statistical analysis

Statistical analyses of clinical variables were performed using SPSS software (version 26.0, IBM, USA). Continuous variables conforming to a normal distribution were expressed as mean ± standard deviation, whereas non-normally distributed variables were expressed as median with interquartile range. Normality was assessed using the Kolmogorov–Smirnov test. Between-group comparisons were performed using the independent-samples t test for normally distributed variables with homogeneous variances and the Mann–Whitney U test otherwise. Categorical variables were summarized as *n* (%) and compared using the chi-square test. A two-sided *P* value < 0.05 was considered statistically significant.

Model discrimination was assessed using the area under the receiver operating characteristic curve (AUC) with 95% confidence intervals. Sensitivity, specificity, accuracy, positive predictive value (PPV), negative predictive value (NPV), F1-score, and balanced accuracy were calculated with AIS as the positive class. Decision curve analysis (DCA) was used to explore the potential clinical net benefit of the models (15). Because individual predicted probabilities were unavailable from the retrospective export, DeLong tests, calibration curves, Brier scores, and formal model-comparison statistics were not calculated; AUC differences were therefore interpreted descriptively (16).

### 2.6 Reporting guidelines

The manuscript was prepared with reference to TRIPOD+AI, CLEAR, and CLAIM reporting principles where applicable to prediction modelling, radiomics, and artificial intelligence in medical imaging (12–14).

## 3 Results

### 3.1 Patient characteristics

The clinical characteristics of the 398 included patients are summarized in Table 1. Compared with the non-AIS group, the AIS group included more men (59.2% vs. 44.3%, *P* = 0.006), smokers (48.0% vs. 27.5%, *P* < 0.001), patients with alcohol history (37.6% vs. 22.3%, *P* = 0.001), and patients with hypertension (76.8% vs. 64.8%, *P* = 0.017). The AIS group also had a higher median ABCD2 score [4 [IQR, 2–5] vs. 2 [IQR, 1–3], *P* < 0.001], systolic blood pressure [144 [126–159] vs. 138 [125–150] mmHg, *P* = 0.023], diastolic blood pressure [84 [75–95] vs. 80 [72–88] mmHg, *P* = 0.001], FPG [6.06 [5.45–8.15] vs. 5.81 [5.27–6.93] mmol/L, *P* = 0.002], and TG [1.56 [1.12–2.10] vs. 1.19 [0.85–1.68] mmol/L, *P* < 0.001], whereas HDL was lower [1.29 [1.10–1.57] vs. 1.38 [1.20–1.60] mmol/L, *P* = 0.037]. TC distributions also differed [4.46 [3.81–5.69] vs. 4.59 [3.89–5.41] mmol/L, *P* = 0.001]. Age, coronary heart disease, diabetes mellitus, and LDL did not differ significantly between groups (all *P* > 0.05). Figure 4 shows the platform-derived importance ranking of the radiomics score and selected clinical features.

**Table 1:** Baseline characteristics of patients in the AIS and non-AIS groups.

Characteristic	AIS (*n* = 125)	Non-AIS (*n* = 273)	*P* value
Age (years)	72.34 ± 10.22	71.28 ± 9.79	0.323
Male sex	74 (59.2%)	121 (44.3%)	0.006
Female sex	51 (40.8%)	152 (55.7%)	
Smoking history, yes	60 (48.0%)	75 (27.5%)	<0.001
Smoking history, no	65 (52.0%)	198 (72.5%)	
Alcohol history, yes	47 (37.6%)	61 (22.3%)	0.001
Alcohol history, no	78 (62.4%)	212 (77.7%)	
Coronary heart disease, yes	21 (16.8%)	56 (20.5%)	0.384
Coronary heart disease, no	104 (83.2%)	217 (79.5%)	
Hypertension, yes	96 (76.8%)	177 (64.8%)	0.017
Hypertension, no	29 (23.2%)	96 (35.2%)	
Diabetes mellitus, yes	47 (37.6%)	96 (35.2%)	0.638
Diabetes mellitus, no	78 (62.4%)	177 (64.8%)	
ABCD2 score	4 (2, 5)	2 (1, 3)	<0.001
Systolic blood pressure (mmHg)	144 (126, 159)	138 (125, 150)	0.023
Diastolic blood pressure (mmHg)	84 (75, 95)	80 (72, 88)	0.001
FPG (mmol/L)	6.06 (5.45, 8.15)	5.81 (5.27, 6.93)	0.002
TC (mmol/L)	4.46 (3.81, 5.69)	4.59 (3.89, 5.41)	0.001
TG (mmol/L)	1.56 (1.12, 2.10)	1.19 (0.85, 1.68)	<0.001
HDL (mmol/L)	1.29 (1.10, 1.57)	1.38 (1.20, 1.60)	0.037
LDL (mmol/L)	2.79 (2.19, 3.49)	2.74 (2.20, 3.38)	0.733

AIS, acute ischemic stroke; FPG, fasting plasma glucose; TC, total cholesterol; TG, triglycerides; HDL, high-density lipoprotein; LDL, low-density lipoprotein.

**Figure 4:**
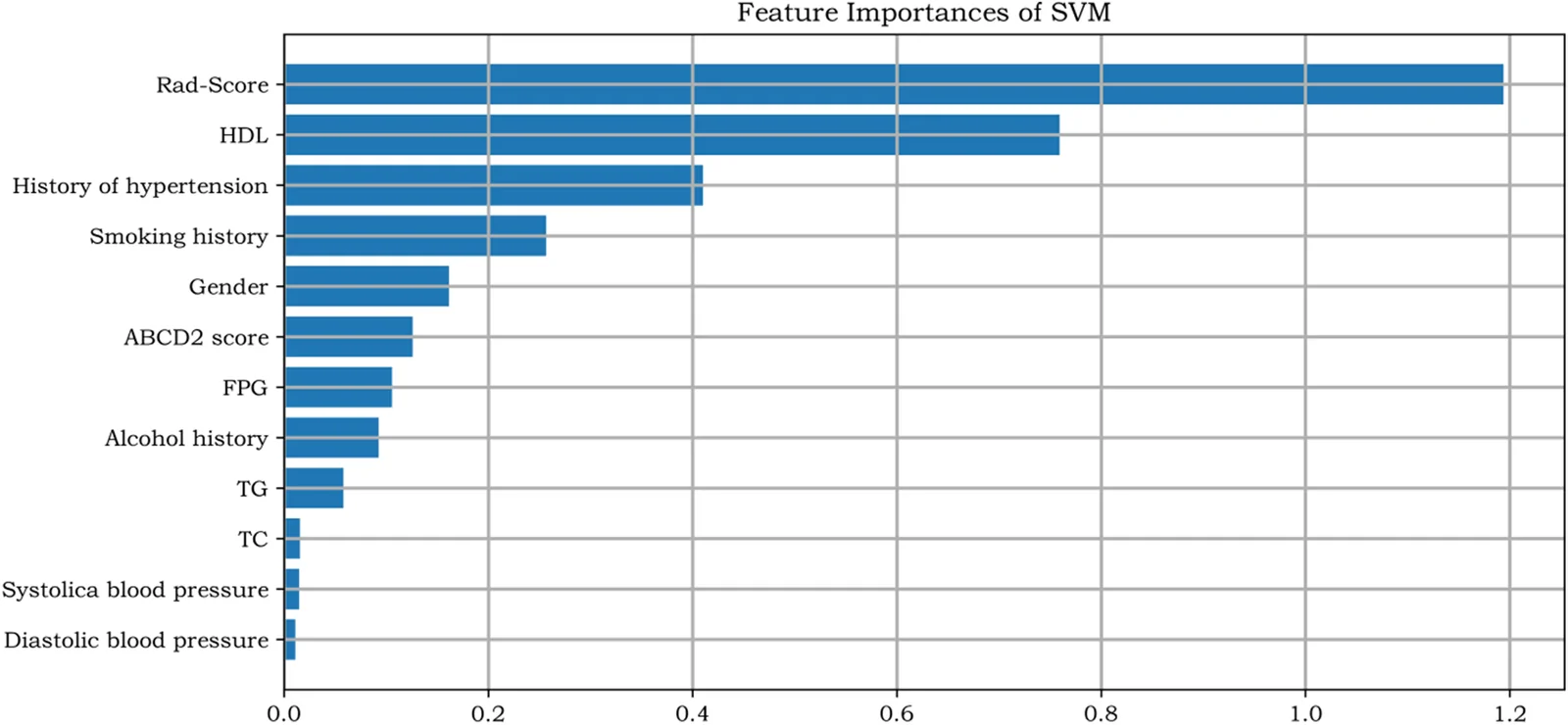
Platform-derived importance ranking of the radiomics score and selected clinical features. Because the feature-importance method depends on the support vector machine implementation, future work should prospectively report whether the ranking is generated using model coefficients, permutation importance, or another method.

### 3.2 Model performance

Using AIS as the positive class, the clinical model showed moderate performance, whereas the radiomics and combined models showed better discrimination. In the training cohort, the combined model achieved the highest AUC (0.900, 95% CI: 0.860–0.940), followed by the radiomics model (0.862, 95% CI: 0.812–0.912) and the clinical model (0.775, 95% CI: 0.720–0.830). The clinical model prioritized sensitivity (0.840) but had lower specificity (0.596), accuracy (0.673), and PPV (0.488), with an NPV of 0.890. The radiomics model had sensitivity of 0.680, specificity of 0.945, accuracy of 0.862, PPV of 0.850, and NPV of 0.866. The combined model had sensitivity of 0.720, specificity of 0.963, accuracy of 0.887, PPV of 0.900, and NPV of 0.882; it also achieved the highest training F1-score (0.800) and balanced accuracy (0.842) (Table 2).

**Table 2:** Performance of the three models in the training cohort with AIS defined as the positive class.

Metric	Clinical model	Radiomics model	Combined model
Sensitivity (AIS)	0.840	0.680	0.720
Specificity	0.596	0.945	0.963
Accuracy	0.673	0.862	0.887
PPV	0.488	0.850	0.900
NPV	0.890	0.866	0.882
F1-score	0.618	0.756	0.800
Balanced accuracy	0.718	0.812	0.842
AUC (95% CI)	0.775 (0.720–0.830)	0.862 (0.812–0.912)	0.900 (0.860–0.940)

AIS was defined as the positive class. AUC, area under the receiver operating characteristic curve; CI, confidence interval; NPV, negative predictive value; PPV, positive predictive value.

In the testing cohort, the radiomics model achieved the highest AUC (0.854, 95% CI: 0.757–0.950), followed by the combined model (0.841, 95% CI: 0.748–0.935) and the clinical model (0.696, 95% CI: 0.568–0.824). The clinical model had sensitivity of 0.560, specificity of 0.836, accuracy of 0.750, PPV of 0.609, and NPV of 0.807. The radiomics model had sensitivity of 0.880, specificity of 0.727, accuracy of 0.775, PPV of 0.595, and NPV of 0.930, with the highest testing F1-score (0.710) and balanced accuracy (0.804). The combined model retained sensitivity of 0.880 but had specificity of 0.673, accuracy of 0.738, PPV of 0.550, and NPV of 0.925. Thus, adding clinical variables did not improve discrimination or overall classification performance over radiomics alone in the internal testing cohort (Table 3). The receiver operating characteristic curves are shown in Figures 5–8. Training-cohort DCA suggested potential net benefit of model-based decision-making; however, because DCA was not available for the testing cohort, the clinical utility findings should be considered exploratory (Figure 9).

**Table 3:** Performance of the three models in the testing cohort with AIS defined as the positive class.

Metric	Clinical model	Radiomics model	Combined model
Sensitivity (AIS)	0.560	0.880	0.880
Specificity	0.836	0.727	0.673
Accuracy	0.750	0.775	0.738
PPV	0.609	0.595	0.550
NPV	0.807	0.930	0.925
F1-score	0.583	0.710	0.677
Balanced accuracy	0.698	0.804	0.776
AUC (95% CI)	0.696 (0.568–0.824)	0.854 (0.757–0.950)	0.841 (0.748–0.935)

AIS was defined as the positive class. AUC, area under the receiver operating characteristic curve; CI, confidence interval; NPV, negative predictive value; PPV, positive predictive value.

**Figure 5:**
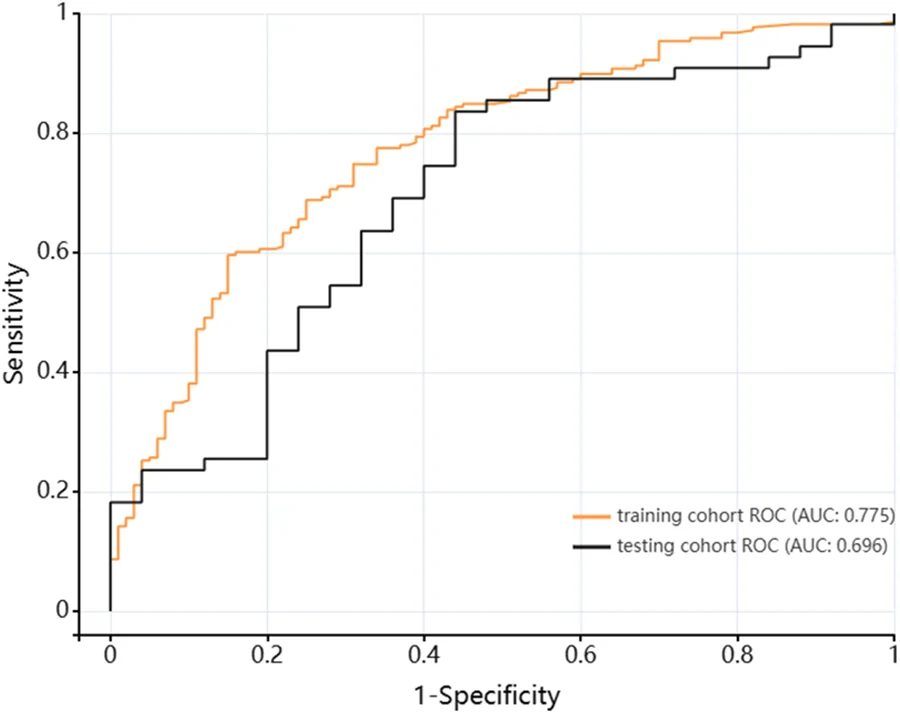
Receiver operating characteristic curves of the clinical-feature-based model for predicting 90-day diffusion-weighted imaging-confirmed acute ischemic stroke after index diffusion-weighted imaging-negative transient ischemic attack. Training cohort: AUC=0.775 (95% CI: 0.720-0.830); testing cohort: AUC=0.696 (95% CI: 0.568-0.824).

**Figure 6:**
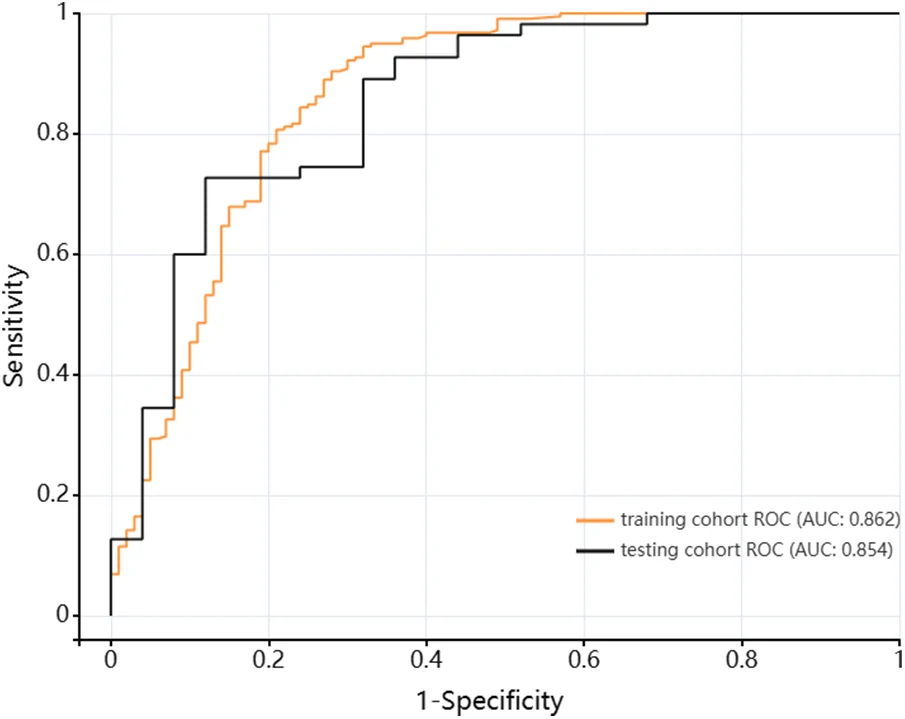
Receiver operating characteristic curves of the radiomics-feature-based model. Training cohort: AUC=0.862 (95% CI: 0.812-0.912); testing cohort: AUC=0.854 (95% CI: 0.757-0.950).

**Figure 7:**
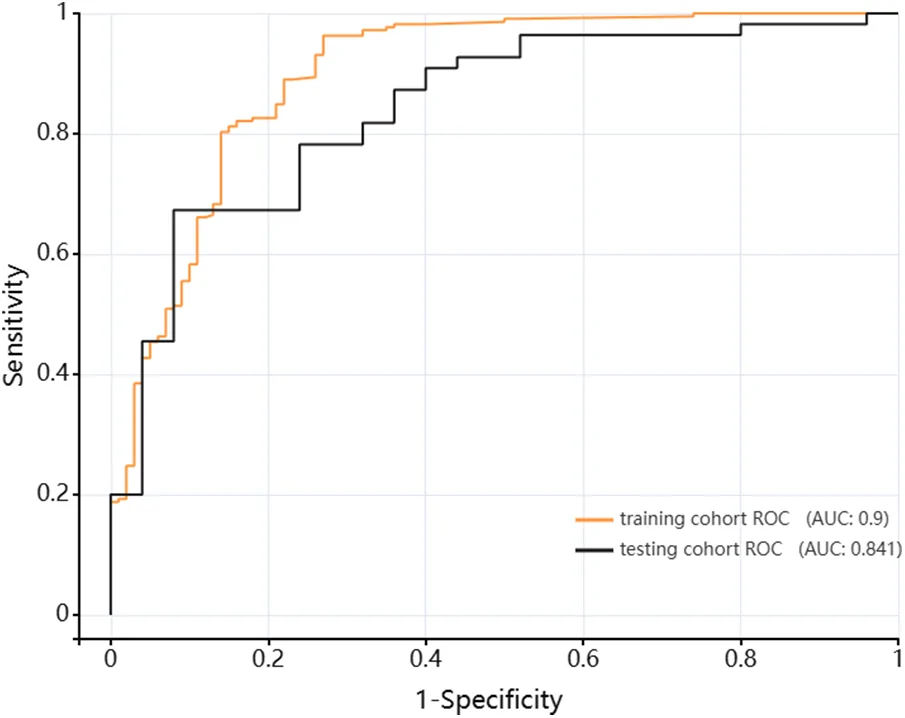
Receiver operating characteristic curves of the combined clinical-radiomics model. Training cohort: AUC=0.900 (95% CI: 0.860-0.940); testing cohort: AUC=0.841 (95% CI: 0.748-0.935).

**Figure 8:**
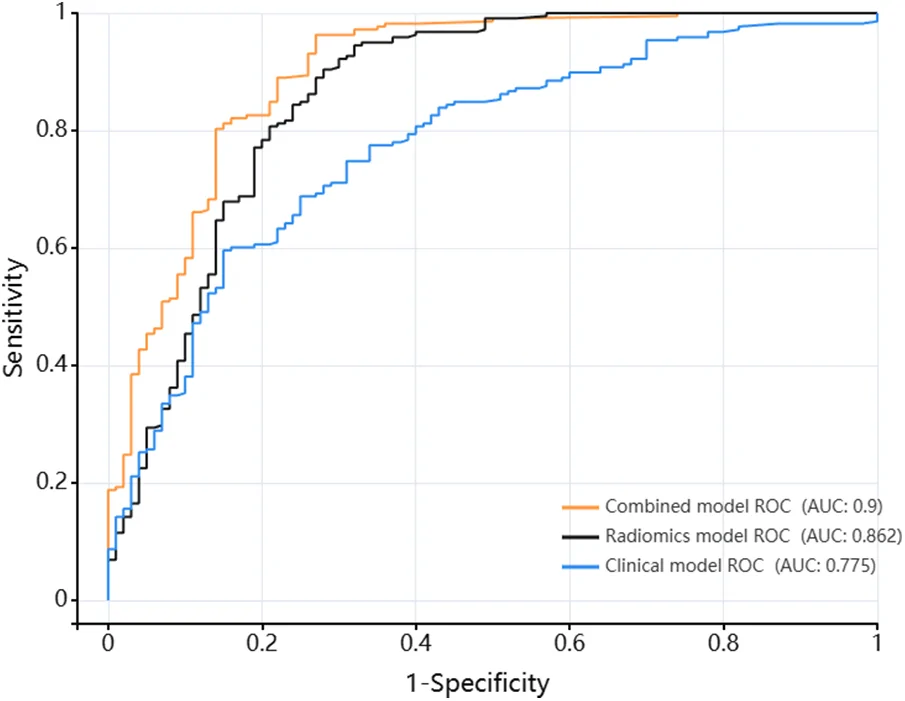
Training-cohort receiver operating characteristic curves comparing the clinical, radiomics, and combined models. The combined model showed the highest AUC in the training cohort, whereas the radiomics model showed the highest AUC in the internal testing cohort.

**Figure 9:**
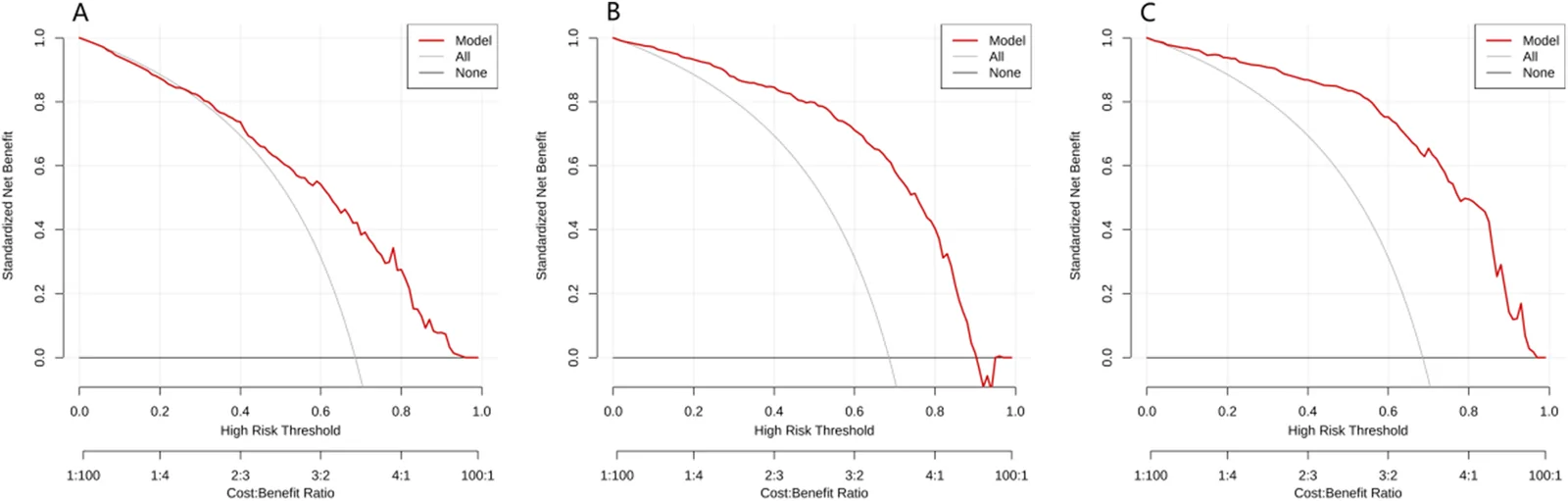
Training-cohort decision curve analysis for the clinical model **(A)**, radiomics model **(B)**, and combined model **(C)** because testing-cohort decision curve analysis was not available, the net-benefit results should be interpreted as exploratory.

## 4 Discussion

In this retrospective study of DWI-negative TIA patients who underwent repeat DWI within 90 days, T2-FLAIR WMH radiomics showed promising internal performance for predicting subsequent DWI-confirmed AIS. The radiomics model outperformed the clinical model in the internal testing cohort, suggesting that quantitative WMH information may add prognostic information beyond conventional clinical variables. At the selected operating point, the radiomics model achieved testing sensitivity of 0.880 and NPV of 0.930, suggesting potential value for identifying patients unlikely to develop DWI-confirmed AIS within this selected cohort; however, its specificity of 0.727 and PPV of 0.595 indicate that positive predictions would still require clinical and vascular evaluation. These prevalence-dependent estimates should not be extrapolated to unselected TIA populations. The combined clinical-radiomics model did not outperform the radiomics-only model in the testing cohort, and therefore the incremental value of adding clinical variables remains uncertain.

The clinical relevance of early risk stratification after TIA is well established. TIA and minor ischemic stroke require prompt etiologic evaluation and secondary prevention, including antithrombotic therapy, lipid management, blood pressure control, diabetes management, smoking cessation, and treatment of large-artery or cardioembolic mechanisms when present (3, 5). In the present cohort, the clustering of male sex, smoking and alcohol histories, hypertension, higher blood pressure, FPG, and TG, and lower HDL in the AIS group is compatible with greater cumulative vascular and metabolic stress, endothelial dysfunction, and impaired cerebrovascular reserve (3, 5, 9). The higher ABCD2 score similarly reflects a greater concentration of established short-term clinical risk features. By contrast, the small TC difference was not directionally consistent with a simple risk gradient and may reflect unmeasured treatment or etiologic heterogeneity; it should not be interpreted causally. Nevertheless, the clinical model had lower testing AUC than the radiomics-based models, supporting the hypothesis that conventional clinical variables alone may not sufficiently capture individual cerebrovascular vulnerability.

WMH are generally interpreted as imaging markers of cerebral small vessel disease and chronic ischemic injury. The radiomics features retained in this study included entropy-, texture-, and wavelet-based features, which may reflect heterogeneity in signal intensity, lesion microstructure, and spatial organization. Such heterogeneity may correspond to varying degrees of demyelination, gliosis, edema, chronic hypoperfusion, and blood-brain barrier dysfunction and may help explain why the radiomics model discriminated better than the clinical model. These interpretations remain hypothesis-generating because radiomics features are mathematical descriptors rather than direct histopathological measurements. The preservation of internal discrimination on routine 1.5 T images is pragmatically encouraging for community and primary hospitals without ready access to 3 T MRI. However, lower signal-to-noise ratio, 6-mm slice thickness, and acquisition on a single scanner may affect texture stability, so the present results do not establish transportability across scanners, vendors, or field strengths.

A key finding is that the combined model had the highest training AUC but did not improve over the radiomics-only model in the testing cohort. Several explanations are possible. First, the addition of multiple clinical variables to a relatively small training set may increase overfitting. Second, some clinical predictors may be redundant with radiomics-derived WMH information or with each other. Third, the selected cohort and high event rate may have affected model calibration and transportability. Therefore, the combined model should not be described as unequivocally superior without further external validation and formal model-comparison testing.

This study has several limitations. First, it was a single-center, retrospective, non-randomized analysis with internal validation only; residual confounding cannot be excluded, and the results require external validation in multicenter prospective cohorts. Second, the requirement for recurrent symptoms and repeat MRI within 90 days introduced selection bias and likely explains the relatively high AIS event rate; thus, the model should not be generalized to unselected TIA populations. Third, the time interval from symptom onset to baseline MRI was not available, and early DWI-negative infarction cannot be completely excluded. Fourth, post-TIA secondary prevention measures, vascular imaging findings, atrial fibrillation status, etiologic stroke subtype, and medication adherence were not consistently available and were not incorporated into the model. These omissions may have affected both model performance and generalizability beyond the study cohort. Fifth, individual predicted probabilities were unavailable from the retrospective platform export, precluding DeLong tests, calibration assessment, Brier score calculation, and testing-cohort DCA. Sixth, detailed radiomics preprocessing settings and SVM hyperparameters were incompletely documented, limiting reproducibility. Finally, conventional WMH markers such as Fazekas score and WMH volume were not directly compared with radiomics features.

Despite these limitations, the findings suggest that T2-FLAIR WMH radiomics may be a useful candidate biomarker for identifying selected DWI-negative TIA patients at risk of short-term AIS. Future studies should prospectively collect standardized imaging, vascular, etiologic, treatment, medication-adherence, cognitive, functional, and follow-up data; perform nested feature selection and hyperparameter tuning; report calibration and external validation; and compare radiomics with Fazekas score, WMH volume, ABCD2, and imaging-enhanced clinical scores. A particularly relevant analysis would compare probable lacunar TIA with non-lacunar TIA using standardized clinical, vascular-imaging, and etiologic criteria, because lacunar and non-lacunar ischemic events differ in risk-factor profiles, pathophysiology, and prognosis (17). Additional priorities include harmonization across 1.5 T and 3 T scanners, assessment of longitudinal WMH change, and evaluation of whether radiomics improves clinical decisions or patient outcomes beyond established care pathways.

In conclusion, these preliminary findings show that a T2-FLAIR WMH radiomics model has promising internal discrimination for predicting 90-day DWI-confirmed AIS after DWI-negative TIA in a selected high-risk cohort. The combined clinical-radiomics model achieved the highest training AUC but did not outperform the radiomics-only model in the testing cohort. Prospective multicenter validation, calibration analysis, external testing, comparison of lacunar and non-lacunar TIA, cross-scanner harmonization, and evaluation of clinical, cognitive, and functional outcomes are required before this approach can be used for routine post-TIA risk stratification.

## Data Availability

The raw data supporting the conclusions of this article will be made available by the authors, without undue reservation.
